# Mrs. Sarah A. Gay

**Published:** 1903-04

**Authors:** 


Mrs. Sarah A. Gay, of Buffalo, widow of Dr. Charles C. F. Gay, died at the family home, February 23, 1903, aged 73 years. Mrs. Gay was the daughter of the late George W. Tifft, and was prominent in social circles for many years.

